# Epidemiology and interactions of Human Immunodeficiency Virus – 1 and *Schistosoma mansoni* in sub-Saharan Africa

**DOI:** 10.1186/2049-9957-2-2

**Published:** 2013-01-24

**Authors:** Humphrey D Mazigo, Fred Nuwaha, Shona Wilson, Safari M Kinung'hi, Domenica Morona, Rebecca Waihenya, Jorg Heukelbach, David W Dunne

**Affiliations:** 1 Department of Medical Parasitology and Entomology, School of Medicine, Catholic University of Health and Allied Sciences, P.O. Box 1464, Mwanza, Tanzania; 2 Department of Environmental Health and Communicable Disease Control, School of Public Health, College of Health Sciences, Makerere University, P.O. Box 7072, Kampala, Uganda; 3 National Institute for Medical Research, Mwanza Research Centre, P.O. Box 1462, Mwanza, Tanzania; 4 Department of Pathology, University of Cambridge, Tennis Court Road, Cambridge, CB2 1QP, UK; 5 Department of Zoology, Faculty of Science, Jomo Kenyatta University of Agriculture and Technology, P.O. Box 62,000, 00200, Nairobi, Kenya; 6 Department of Community Health, School of Medicine, Federal University of Ceará, Ceará, Brazil

**Keywords:** *Schistosoma mansoni*, HIV-1, Co-infections, Immunological interactions, Deworming

## Abstract

Human Immunodeficiency Virus-1/AIDS and *Schistosoma mansoni* are widespread in sub-Saharan Africa and co-infection occurs commonly. Since the early 1990s, it has been suggested that the two infections may interact and potentiate the effects of each other within co-infected human hosts. Indeed, *S. mansoni* infection has been suggested to be a risk factor for HIV transmission and progression in Africa. If so, it would follow that mass deworming could have beneficial effects on HIV-1 transmission dynamics. The epidemiology of HIV in African countries is changing, shifting from urban to rural areas where the prevalence of *Schistosoma mansoni* is high and public health services are deficient. On the other side, the consequent pathogenesis of HIV-1/*S. mansoni* co-infection remains unknown. Here we give an account of the epidemiology of HIV-1 and *S. mansoni*, discuss co-infection and possible biological causal relationships between the two infections, and the potential impact of praziquantel treatment on HIV-1 viral loads, CD4^+^ counts and CD4^+^/CD8^+^ ratio. Our review of the available literature indicates that there is evidence to support the hypothesis that *S. mansoni* infections can influence the replication of the HIV-1, cell-to-cell transmission, as well as increase HIV progression as measured by reduced CD4^+^ T lymphocytes counts. If so, then deworming of HIV positive individuals living in endemic areas may impact on HIV-1 viral loads and CD4^+^ T lymphocyte counts.

## Multilingual abstracts

Please see Additional file 1 for translations of the abstract into the six official working languages of the United Nations.

## Review

### Introduction

Worldwide, HIV-1 infections remain a major public health problem. In 2010, over 31 million adult individuals (>15 years) were living with the disease and new cases of the disease were estimated to be at 2.7 million individuals [1]. The sub-Saharan African region continues to carry the largest proportion of the global disease burden [1]. In 2010, over 68% of global cases of HIV were in sub-Saharan Africa [1]. In this region, an estimated 1.9 million individuals were newly infected with HIV during 2010, comprising about 70% of all new cases of the disease worldwide [1]. However, in the East African region, the HIV epidemic has started to decline and has stabilized in some areas [2]. The national prevalence of HIV varies among countries in the region, from 3% in Rwanda, 5.8% in Tanzania, 6% in Kenya to 6-7% in Uganda [2,3]. The risk factors for HIV transmission in sub-Saharan Africa vary dramatically across sub-populations with different demographic characteristics [4,5]. The key risk factors for heterosexual transmission of HIV in Africa are commercial sex (prostitution), high population mobility, concurrent or multiple partners or number of lifetime sexual partners, residential location (rural *versus* urban), history of active or passive sexually-transmitted disease and lack of male circumcision [4,5]. Several epidemiological studies have reported vulnerable groups such as female bar workers [6], female commercial sex workers, long-distance truck drivers and their partners [7]. Fishing communities remain at higher risk of acquiring and transmitting HIV, and play a key role in the spread of HIV and in the maintenance of the HIV infection levels in the population [8,9].

Schistosomiasis is a chronic, water-borne helminth disease, endemic in Africa for many centuries [10-12]. The current global estimate indicates that 779 million people in 76 countries are at risk for schistosomiasis and that 207 millions are infected [13]. Approximately 120 million people have schistosomiasis-related symptoms and 20 million suffer from the chronic form of the disease [14]. In Africa, urogenital schistosomiasis, caused by infection with *Schistosoma haematobium*, and intestinal schistosomiasis caused by *S. intercalatum* and *S. mansoni*, are highly endemic [13]. However, *S. haematobium* and *S. mansoni* are the most widely distributed causing the greatest burden of mortality. A recent meta-analysis of existing data suggests that up to 280,000 deaths annually are related to schistosomiasis (both urogenital and intestinal) in sub-Saharan Africa [15,16]. The disability-adjusted life years (DALYs) lost due to schistosomiasis are estimated at 4.5 million [16]. However, not all authors agree with this estimate, some arguing that it is an underestimation of the real impact of schistosomiasis [15,17]. There is a real risk that, despite effort to control schistosomiasis, the global prevalence of schistosomiasis may still increase due to the effects of increasing numbers of agricultural irrigation schemes, constructions of dams and man-made lakes for hydroelectric power generation, as well as civil wars, which contribute to increased human population movements [13,18].

In established endemic areas, *S. mansoni* affects individuals of all age groups, but the prevalence and infection intensity is usually seen to peak among children under 15 years of age [19,20]. This age-pattern of infection intensity has been reported to develop within 2 years amongst immigrants newly exposed to infection on migration into *S. mansoni* endemic areas [21]. Although age-specific behavioral patterns, with high water contact and exposure to infection, often favour greater childhood infection intensities, the slow development of a partial immunity in endemic area residents may contribute to the lower infection intensities observed in adults [22,23]. However, high occupational exposure, associated with fishing for example, can result in maintenance of high intensities of infection into adulthood [24].

**Figure 1:**
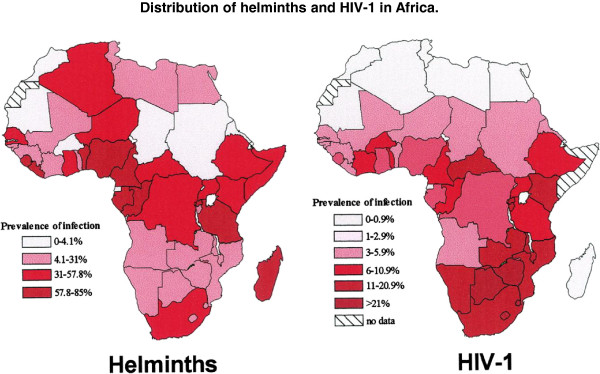
**Distribution of helminths and HIV-1 in Africa **[26].

Human Immunodeficiency Virus-1 and *S. mansoni* infections are co-endemic in Sub-Saharan Africa and co-infection occurs in highly endemic areas (Figure 1) [25,26]. In the early 1990s, it was hypothesized that helminth infections in sub-Saharan Africa were associated with a high transmission of HIV [25,26] and a faster progression of HIV to AIDS [27-42]. In HIV co-infected individuals, helminth infections may cause general immune activation and affect the pattern of cytokine secretions [25-42]. Further effects observed in HIV-1 – helminth co-infected individuals included the modulation of the immune response against helminths [28,29,43], an impaired schistosome egg excretion [27,31] and increased HIV-1 viral loads after chemotherapeutic deworming [32-42]. The immunological effects and morbidities associated with helminth infections observed in HIV-1 positives individuals vary with the species of helminth involved. Some effects are common to all helminths, others are specific to particular helminth species such as schistosomes. Of the schistosomes that infect man in sub-Saharan Africa, *S. haematobium,* causing urogenital schistosomiasis, presents a distinct and potentially important specific risk factor for the acquisition of HIV-1/AIDS via urogenital tract lesions, and this has been reviewed elsewhere [44-46]. In contrast, *S. mansoni*, which is the focus of this review, rarely causes genital lesions but may still act as a risk factor for HIV-1 transmission and progression of the disease through within-host interactions with HIV-1. Thus, here we review the evidence for biological causal effects of HIV-1/AIDS and *S. mansoni* in human hosts. Specifically, we focus on immunological interactions (bi-directional effects in causing morbidities), the efficacy of anti-schistosome chemotherapy, and its effect on HIV-1 related parameters (such as HIV-1 viral loads and CD4^+^ lymphocytes) in HIV-1- *S. mansoni* co-infected individuals. 

### Methodology

Data for this review were collected by searches in NCBI PubMed, EMBASE, Global Health and from the references of published manuscripts, relevant articles and doctoral theses. For computerized databases, the search strategy included only permutations of key terms, which were relevant to the study. Initially, all searches began with the text string “schistosom*” and specified keywords in permutations related to co-infection, including “HIV”, “HIV/AIDS”, “Viral loads”, “co-infection”, “deworming”, “treatment”, “intestinal schistosomiasis”, “*Schistosoma mansoni*”, “*Schistosoma haematobium*”, “urinary schistosomiasis”, “soil-transmitted helminths” and “filarial”. In addition, we reviewed the past and current available publications on WHO, UNAIDS and UNICEF websites, which were relevant to our review.

### Characteristics of the immune response to helminth infections

Most of the human parasitic nematodes and trematodes that occur in sub-Saharan Africa have complex multi-stage lifecycles involving one or two host species and a complex immune modulation response in their hosts [46-50]. The available evidence indicates that the immune responses of various hosts (both natural infections and experimental models) to helminth infection are similar, being commonly characterized by type 2 T-helper lymphocytes with production of significant quantities of interleukin-4, IL-5, IL-9, IL-10 and IL-13 [46-50]. The increased level of Th_2_ interleukins are accompanied by an increased production of immunoglobulin E (IgE), eosinophils and mast cells responses [47,48]. In early helminth infections, the infectious larval stages induce the production of either Th_1_ or Th_2_ cytokines response from T-lymphocytes and other immune cells [49]. In *S. mansoni* infections, the cercaria infective stage stimulates Th_1_ immune responses during the early phase of the infection in mouse models. In chronic human and experimental infections, *Schistosoma mansoni*, like all helminth infections, induces a predominantly Th_2_ immune response, characterized by interleukin-4, IL-5, IL-9, IL-10 and IL-13, antibody (IgE and IgG4), eosinophils and mast cells. However, these responses are tightly regulated to produce a modified Th_2_ immune environment. The down-regulatory mechanisms involved not only down-regulate Th_2_, but also the Th_1_ mediated responses that have been observed, in both mice [47,48] and humans [49,50] when exposed to heavy, primary, schistosome infections.

### Immunological interactions of Human Immunodeficiency-1 and *Schistosoma mansoni*

#### Potential immunological interactions

The major hallmark of HIV-1 infections includes the destruction or depletion of the total body of helper CD4^+^ T-lymphocytes, both naïve (CD45RA^+^) and memory cells (CD4RO^+^), and a subsequent loss of immune competence [26,51]. Studies have indicated that destruction of the CD4^+^ cell pool increases susceptibility of the host to other infectious diseases [26]. Earlier studies demonstrated a correlation of maintaining the Th1 (CD8^+^- T-lymphocytes) immune profile and slow progression of the HIV-1 infections [52-56]. During this phase, uncontrolled replication of the HIV-1 infections leads to activations of the CD8^+^ T-lymphocytes (those expressing CD38, CD45RO and HLA-DR) [57-60] and increased concentration of cytokines such as IFN-α, TNF-γ and IL-1β [52,57-60]. A switch of the immune response from Th1 (CD8^+^ T-lymphocytes) to Th2 (CD4^+^ T-lymphocytes) with subsequent production of its associated cytokines are related to fast progression of the disease and chronic activation of the immune responses [52,55]. The proliferations of the Th2-CD4^+^ is also associated with the proliferations of other related CD4^+^ regulatory subsets such as CD4^+^ CD25^+^ (CD4^+^ T_reg_) which have been shown to express Cytotoxic T lymphocyte-associated antigen 4 (CTLA-4) that exerts an inhibitory effect on T cell proliferation by secreting immunosuppressive cytokines such as TGF-β and IL-10 [60-65]. The TFG-β plays a pivotal role in CD4^+^ T-cell regulation by inhibiting its proliferation and acquisition of the effectors function by the naïve T-cells [66]. Increased CTLA- 4 expression correlates with markers of HIV disease progression and the up-regulation of the CTLA- 4 also increases CCR5 expression on the surfaces of CD4^+^ T-lymphocytes which enhance the susceptibility of these cells to HIV-1 infections and cell to cell HIV transmission [67]. Studies have indicated that destruction of the CD4^+^ cell pool by HIV-1 infection increases susceptibility of the host to other infectious diseases [26]. These immune characteristics of HIV-1 and *S. mansoni* infections clearly give rise to the potential for a number of immunological one-way and two-way modulating interactions between them in co-infected populations.

The CD4^+^ T-helper lymphocytes responses are central to the development of immunopathology in *S. mansoni* infections [68]. The chronic phase, which is characterized by the production of soluble egg antigens (SEA), the inflammatory cytokines response to SEA are significantly reduced and the Th2 response is characterized by up-regulation of IL-10 and TGF-β which down-regulates the production and effectors functions of the Th1 response [69,70]. The release of these cytokines leads to the productions of the IgG4, suppressed parasites-specific T cell proliferation, reduced level of Th2 and Th1 cytokines [71]. At this stage only few *S. mansoni* infected individuals develop severe hepatosplenic disease characterized with development of granulomas [71-74]. The resulting T- dependent granulomas protect host tissues from egg-produced antigens [75]. The granulomas around the trapped eggs are composed of collagen fibers and inflammatory cells of Th_2_ origins, including eosinophils, macrophages and CD4^+^ T-cells [74]. In addition, the mechanisms responsible for granuloma formation are also involved in the process of *S. mansoni* eggs excretion [76]. In fact, individuals with chronic *S. mansoni* pathologies express high level of Th1 and Th17 responses which lead to inflammation and fibrosis around the deposited schistosomes eggs in tissues [71].

#### Effects of HIV-1 on *Schistosoma mansoni*

The destruction of helper CD4^+^ T-lymphocytes by the HIV-1 virus in co-infected individuals could affect granuloma formation, and alter egg excretion efficiency. Studies on immuno-suppression animal models have demonstrated that the excretion of *S. mansoni* eggs is immune dependent, and that T-cells, specifically the anti-egg Th_2_ responses [77] are necessary for the transposition and excretion of eggs from the host blood stream into the intestinal lumen [78,79] as well as the development of granuloma [80,81]. In Kenya, Karanja *et al.*[82] demonstrated that HIV infected individuals had a reduced eggs excretion of *S. mansoni,* correlated with decreased CD4^+^ T lymphocytes counts [82]. Similar observations on the reduced eggs excretion were reported in HIV-1 positive individuals co-infected with *S. mansoni* and *S. haematobium* in Ethiopia, Zambia and Congo [25,82,83]. In rural Zimbabwe, although the study was limited by low infection intensities of *S. mansoni*, no association was demonstrated between the HIV-1 status or CD4^+^ T lymphocytes counts and the eggs excretion efficiency [27]. Based on these observations on the CD4^+^ T-lymphocytes response during *S. mansoni* infection in multiple animal models and human studies, could lead to greater numbers of eggs retained in the host’s affected body organs. If this hypothesis is correct, then HIV-1 positive individuals co-infected with *S. mansoni* might have altered morbidity (i.e. less fibrous material formation at the granuloma area, or inability to contain the egg released antigens) than individuals infected with *S. mansoni* alone*.* This could have a significant bearing on the parasitological diagnosis of the infection, which is dependent on detection of excreted parasite eggs [30].

The potential for HIV to affect *S. mansoni* egg excretion not only potentially affects parasitic diagnosis of infection, but also co-infection studies, this makes the detection of circulating schistosome antigens released by in situ worms particularly important in detecting and, to some extent quantifying, these infections. Circulating Cathodic Antigens (CCA) and Circulating Anodic Antigen (CAA) are *S. mansoni* related gut antigens which are regurgitated by the adult and juveniles stages with the by-products of host red blood cell digestion. CCA can now be detected by antibody-based rapid diagnostic tests in urine samples [42,84]. Such antigen-detection tests have many advantages, including demonstration of active infections of *S. mansoni* in the absence of detectable egg excretion, the effects of treatment and in term of diagnosis it has high specificity [42,84]. When CAA/CCA positivity has been employed as a diagnostic criterion for *S. mansoni* infection after praziquantel treatment in HIV-1 and *S. mansoni* co-infected individuals compared with those with only *S. mansoni* infections. The result showed a lower clearance rate of the adult worms in treated HIV-1 positive individuals co-infected with *S. mansoni*[41]. This observation was inconsistent with the results of Karanja *et al.*[40], who identified equally decreased levels of CCA following praziquantel therapy in individuals who were HIV-1 positive and co-infected with *S. mansoni* as compared to individuals with *S. mansoni* infection only [41]. It was argued that the difference in *S. mansoni* intensity of infection between the two study populations and the dominance of *S. haematobium* in the study of Kallestrup *et al.*[41] could have contributed to the discordance between those studies’ results. The discrepancy observed between these studies calls for further studies to elucidate efficacy of praziquantel in HIV-1 infected individuals co-infected with *S. mansoni*.

Granuloma formation in *S. mansoni* infection is a CD4^+^ dependent process and earlier studies have hypothesized that the destruction of helper CD4^+^ T-lymphocytes (Th_2_) by HIV-1, coupled with the significant importance of CD4^+^ cells in the formation of granuloma, may lead to a decreased ability of the Th_2_ arm to produce pro-inflammatory cytokines such as TNF-α, IL-6, IL-1, IL-13 which are responsible for stimulating the inflammatory cells responsible for fibrogenesis, and hence lead to severe hepatic morbidity [78,85]. Immunological studies have demonstrated that, T-cells from the peripheral blood of HIV-1 positive individuals co-infected with *S. mansoni* responded to egg antigens by producing less IL-4 and IL-10 and a lower amount of IFN-γ as compared to those from individuals infected with *S. mansoni* alone*,* indicating immune skewing from Th_2_ to Th_1_[78]. Supportive evidence from animal models indicates that, granuloma formation is severely restricted in immuno-deficiency mouse models [80,81]. It is possible that granuloma formation may help to contain hepatotoxins (omega-1-ribonuclease and IPSE/alpha-1) that are released from *S. mansoni* eggs trapped in the liver [86-88] which may increase the risk of liver parenchyma damage and cause severe necrosis [75,76,89].

A community-based study in fishing communities in northwest Tanzania, showed that there were no differences in the distribution of *S. mansoni* related morbidities, as detected by ultrasonography, between HIV co-infected individuals with *S. mansoni* and those with single infection of *S. mansoni*[79]. This study though did not measure the CD4^+^ counts of the HIV-1^+^ positive study participants co-infected with *S. mansoni.* Similarly, in Kenya, there were no significant differences in the distribution of ultrasound-detectable pathology (hepatomegaly, splenomegaly, hepatic fibrosis, periportal fibrosis and gallbladder wall thickness) in HIV-1 positive individuals co-infected with *S. mansoni* as compared with HIV-1 negative individuals infected with *S. mansoni*[43]. The study demonstrated that hepatic fibrosis, in the absence of severe hepatosplenomegaly, was associated with a significant decrease in CD4^+^ T-lymphocytes in HIV-1 negative individuals infected with *S. mansoni,* and that the decrease correlated with increasing grade of liver fibrosis [43]. In HIV-1 positive individuals co-infected with *S. mansoni,* reduced CD4^+^ T-cells counts levels did not necessarily imply the development of severe hepatic morbidities or altered patterns of hepatic fibrosis due to the effects of hepatotoxins [43]. In addition, there was no difference in the level of measurable fibrosis and the level of liver parenchyma damage as measured by the levels of circulating liver enzymes (glutamic oxaloacetic transaminase and aspartate aminotransferase) in individuals with HIV-1 co-infection as compared with HIV-1 negative individuals infected with *S. mansoni*[43]. Importantly, there were no significant correlations between CD4^+^ T cells count and circulating liver enzyme levels in HIV-1 positive individuals co-infected with *S. mansoni* or in HIV-1 negative individuals infected with *S. mansoni*[43]*.* However, it is worth noting that the passive transfer of specific anti-omega 1 antibody is sufficient to completely prevent hepatocyte damage in *S. mansoni* infected immunosuppressed mice that have severely impaired anti-egg granuloma [90]. Anti-omega 1 antibodies were not assayed in the human studies [43]. The observation that hepatic fibrosis is associated with reduced CD4^+^ T-lymphocytes in HIV-1 positive and negative individuals implies that *S. mansoni* associated liver pathologies could speed up the progression of HIV to AIDS through the depletion of CD4^+^ T cells in co-infected individuals [43]. It could also be speculated that, the hyporesponsiveness of the T-cells due to chronic activations of the immune system and differentiations of the T_regs_ and released of TGF-β and IL-10 could in part explains the low CD4^+^ counts observed in HIV-1 positive and HIV-1 negative individuals infected with *S. mansoni*. However, this observation calls for further studies to confirm the observation.

#### Effects of *Schistosoma mansoni* on HIV-1

*Schistosoma mansoni* infections induce an immune modulation, which shifts from T-helper 1 to predominant T-helper 2 cytokines [69,70,91]. The cytokines associated with T-helper 2 lymphocytes down-regulate the cytotoxic effects of T-cytotoxic (CD8^+^) lymphocytes which are essential for the initial control of viral replication [69,70,81,91]. In animal studies, mice co-infected with *S. mansoni* and vaccinia virus expressing the HIV envelope displayed a shift towards a Th_2_ response which down-regulated Th_1_ cytokines production and impaired the cytotoxic effects of CD8^+^ on the virus [81,82]. In addition, an increase in viral replication and the alteration of T-cells subsets have been observed in Rhesus Macaque monkeys co-infected with *S. mansoni*[92]. In human studies, HIV-1 positive individuals co-infected with *S. mansoni* in western Kenya demonstrated an alteration of the immune response to *S. mansoni* characterized by a low level of IL-4 and IL-10 production [78].

*In vitro* studies on human peripheral blood mononuclear cells from individuals infected with schistosomiasis have shown an increased susceptibility of these cells to HIV-1 as compared to helminth free individuals [31]. The expression of the chemokine receptors CCR5 and CXCR4 on the surfaces of CD4^+^ T-lymphocytes, which have been stimulated by Th_2_ cytokines, make these cells more susceptible to HIV-1 infection [31,32]. In fact, these receptors serve as co-receptors for HIV-1 entry into the cells [31,32]. In Kenya, individuals infected with schistosomiasis expressed higher cell surface densities of these receptors as compared to individuals cured of the disease [31]. These observations imply that HIV-1 replication proceeds more rapidly in activated T cells, especially in those with Th_2_ or Th_0_ phenotypes [32].

Individuals co-infected with HIV-1 and *S. mansoni* may have reduced ability to mount potent protective immune responses against a number of viral infections. Similarly, individuals co-infected with chronic Hepatitis C virus (HCV) and *S. mansoni* demonstrated a decreased HCV- specific CD4^+^ T cell proliferative response as compared with individuals with HCV alone [33]. In Uganda, concomitant infections of *S. mansoni* and HIV-1 was associated with decreased Gag-specific cytolytic (CD8^+^) responses, showing an alteration of the effectors functions of HIV infection attributed to schistosomiasis [34]. Moreover, the detection of Gag-specific positive CD8^+^ T cells in co-infected individuals shows that *S. mansoni* may be responsible for the modulation of the cellular immune response to HIV [34]. T-regulatory cells are an important component of regulation of T cell activation. It has been reported that an expansion in T-regulatory cells (T-reg) occurs during the chronic phase of HIV infection. There is some debate as to whether the expansion of T-reg numbers is detrimental, due to suppression of cellular mediated immunity, or beneficial, due to limiting cellular activation, and therefore co-receptor expression and targets for HIV-1 infection [93]. *Schistosoma mansoni* infection was found to expand the proportion of circulating CD25hi CD4^+^ cells, a significant proportion of which are likely to be FoxP3^+^ve T-reg cells, amongst sand-harvesters in Kisumu, Kenya [94]. However, no significant difference in the proportion of CD4^+^CD25hi cells was observed between individuals who were sero-positive and negative for HIV-1 [94]. As the role of T-regs in HIV infection is clarified [52,53], further studies using a wider range of T-reg markers such as FoxP3^+^ve and CD127lo [95,96] will be required to determine whether this important sub-type is affected by co-infection with *S. mansoni*.

Evidence suggests that the expression of the co-receptors on activated CD4^+^ T lymphocytes increases the susceptibility of these cells to HIV infection in HIV-uninfected populations, and may also speed up the progression of HIV to AIDS by increasing plasma viral loads and decreasing CD4^+^ T-lymphocytes in co-infected individuals [31,32]. Increased HIV plasma viral loads determine disease progression and risk for HIV transmission in between partners [35]. However, most cross-sectional and other observational studies have failed to provide evidence that decreased CD4^+^ T cells and increased HIV-1 viral loads is associated with heavy helminth infections [29,36,37]. Previous authors have suggested that helminth infection intensity could in part contribute to decreased CD4^+^ T cell counts or speed up the progression of HIV-1 to AIDS, meaning that HIV-1 positive individuals with higher *S. mansoni* infection intensity could have reduced CD4^+^ T cells counts and higher HIV-1 viral loads. Similar findings on the other helminth species have been reported [83]. There is however no evidence on the linear relationship between *S. mansoni* infection intensity and HIV-1 viral loads in co-infected individuals and this calls for further studies.

#### Efficacy of praziquantel treatment of *Schistosoma mansoni* in HIV-1 co-infected individuals

In the last three decades, praziquantel has been the drug of choice for treatment of schistosomiasis, especially in sub-Saharan Africa, because of its high level of efficacy against all schistosome species, its ease of administration and lack of serious adverse effects [97]. A single dose of 40mg/kg body weight is reported to result into human schistosomiasis cure rates that vary widely (60% - 90%) between different studies, but which consistently result in reductions of infection intensity of more than 95% [23,98]. The efficacy of praziquantel depends to some extend on its synergy with an intact immune response, studies in immunodeficient animal models have demonstrated reduced efficacy of praziquantel [98-101]. In addition, praziquantel efficacy can be increased by pre-immunization of mice with schistosome antigens, whereby the generated parasite-specific antibodies increase praziquantel efficacy [78,79]. Several studies have been carried out in HIV-1 positive individuals to test whether or not the efficacy of praziquantel has an immune dependent component in human-*S. mansoni* infections, as this has been reported in mice [40-42].

In western Kenya, a study was carried out involving male car-washers (>18 years) in which 15 individuals were co-infected with *S. mansoni* and HIV-1 and 32 individuals had *S. mansoni* infection alone [40]. Majority of these individuals had heavy infections of *S. mansoni*. The results of that study indicated that HIV-1 sero-positivity status did not affect the efficacy of praziquantel. In the treated groups (HIV-1 positive co-infected with *S. mansoni versus* HIV-1 negative infected with *S. mansoni*), a single dose of praziquantel resulted in a >93% reduction in *S. mansoni* infection intensity regardless of HIV-1 serostatus and percentage of CD4^+^ T- cell [40]. Similarly, a prospective cohort study in Zambia which included individuals aged 10 – 55 years, in which 47 were co-infected with HIV-1 and *S. haematobium* and 335 HIV-1 negative individuals infected with *S. haematobium* demonstrated that praziquantel treatment resulted in 99.81% reduction in average infection intensity, despite a concurrent HIV-1 infection [42].

#### Effects of praziquantel treatment of *S. mansoni* on HIV-1 viral loads and CD4^+^ T-lymphocytes levels in co-infected individuals

The individual immunological interactions between these two common pathogens suggest that increased expression of Th_2_ cytokines caused by *S. mansoni,* raising the possibly that co-infection may increase HIV replication and cell-to-cell transmission, as well as increase the rate of HIV progression, as measured by reduced CD4^+^ T lymphocyte counts [102]. If so, then deworming of HIV positive individuals living in endemic areas may reduce the HIV-1 viral loads and increase both the CD4^+^ T lymphocyte counts immune responsiveness of the T-cells to both HIV-1 and *S. mansoni* infections [103-107].

Recent data from randomized control trials and observational studies have highlighted decreased HIV-1 viral loads, decreased expression of co-receptors CCR5 and CXCR4 on the surface of CD4^+^ T lymphocytes and improved CD4^+^ T lymphocytes counts, following the treatment of various species of helminths [31,103-106,108-111]. Praziquantel treatment in *S. mansoni* infected Kenyan car washers resulted in a drop of CCR5 and CXCR4 levels expressed on the CD4^+^ T lymphocytes in both HIV-1 infected and un-infected individuals, suggesting that the treatment of individuals infected with *S. mansoni* alone*,* or co-infected with HIV-1 and *S. mansoni*, could decrease the risk of HIV transmission to individuals with schistosomiasis, or intercellular transmission in HIV-1 infected individuals [31]. In rural Zimbabwe, praziquantel treatment of HIV-1 co-infected individuals resulted in the decline of HIV-1 viral loads in the group that received immediate treatment as compared with those who received treatment three months later [109]. In addition, despite an observed increase in HIV-1 viral loads after three months, the mean HIV-1 viral loads in the early treatment group was lower than in the delayed treatment group [109]. However, subsequent studies did not observe any impact of anthelminthic treatment and decline in HIV-1 viral loads, lower CD4^+^ T lymphocytes or faster progression to AIDS [37,39,110]. In Uganda, a significant transient increase in HIV-1 viral loads and a decrease in CD4^+^ T lymphocytes were observed one month post-praziquantel treatment in HIV-1- *S. mansoni* co-infected individuals [37,111]. The mechanisms that might favour increased viral loads and decreased CD4^+^ T lymphocytes remain unclear and open up a number of interpretations. It is possible that the adult worm death following praziquantel treatment provides an antigenic stimulation that increases Th_2_ activation and hence increases HIV-1 replication [31,102,112,113]. Alternatively, anthelminthic treatment may suppress the production of inflammatory and anti-inflammatory cytokines that are maintained by chronic helminth infections [113].

Increased levels of CD4^+^ T lymphocytes in HIV-1 positive individuals co-infected with *S. mansoni* have been reported after praziquantel treatment [109]. In rural Zimbabwe, a randomized control trial reported increased CD4^+^ T lymphocyte counts in those receiving praziquantel treatments, as compared to those treated three months later [108]. However, this observation contrasted with other similar studies that reported decreased CD4^+^ T lymphocyte counts after praziquantel treatment [36,37,39,110]. The lack of consistency between these studies calls for further studies to clear the observed discrepancies.

#### Effects of praziquantel treatment on hepatic morbidities in co-infected individuals

The intestinal form of schistosomiasis mansoni is characterized by abdominal pain, diarrhea, bloody stool, nausea, fatigue and drowsiness. *S. mansoni* eggs that do not penetrate the gut wall can pass via the portal vein to be trapped in the liver tissues where they provoke vascular, inflammatory and granulomatous changes [114]. In severe advanced cases, this can lead to hepatosplenomegaly and portal hypertension, which can lead to development of oesophageal varices, ascites and risk of haematemesis [114]. Autopsy studies have associated severe hepatosplenic disease and portal hypertension with gross hepatic periportal fibrosis [115,116].

The impact of praziquantel treatment on *S. mansoni* is not only evaluated in terms of reduction in the infection intensity but also in terms of the reversibility of hepatosplenic morbidities after treatment [97,117-121]. Not all *S. mansoni* associated hepatosplenic morbidities reverse after treatment, mild or low grade periportal fibrosis can be seen to reverse 12 months after treatment, but advanced periportal fibrosis is considered to be irreversible and studies have reported the progression of organomegaly (hepatomegaly or splenomegaly to hepatosplenomegaly) after treatment [121,122].

Based on the reduced anti-egg granulomatous response and fibrosis [75,76,89] and the reduced efficacy of praziquantel treatment, reported in immunosuppressed mice [98-101], it is possible that human HIV-1 infection could impact on co-infecting schistosome morbidity, including regression or progression of hepatic morbidity after praziquantel treatment. As yet, only one human ultrasound study of HIV-1 and *S. mansoni* co-infection hepatic morbidity has been reported [43]. To date, no single study has evaluated the impact of praziquantel treatment on hepatic and splenic morbidities in individuals co-infected with *S. mansoni* and HIV-1. This calls for further studies on large sample sizes to understand the liver and spleen morbidities in individuals co-infected with HIV-1 and *S. mansoni*.

#### Possible implications of HIV-1 and *S. mansoni* co-infection on Mass Drug Administration

Based on the evidence above on the interactions between HIV-1 and *S. mansoni* in sub-Saharan Africa and the fact that the two infections are highly prevalent, co-infections with the two diseases in high risk groups such as fishing communities are possibly high and their interactions could be the cause of the severe morbidities observed and high prevalence of HIV-1. Despite the potential risks related to co-infection, little is known of the interaction between these infections in high-risk communities [70]. In the sub-Saharan Africa, the control of schistosomiasis focuses mainly on the reduction of morbidities and mainly targets groups at risk [97]. Control approaches often involve Mass Drug Administration (MDA) of praziquantel to treat schistosomiasis, combined with albendazole to treat nematode infections, to schoolchildren or through child health clinics [97]. Occasionally, MDA is preceded by mass screening of schistosomiasis before treatment for the purpose of monitoring the impact of MDA on targeted infections [97]. Evidence from clinical studies suggests that de-worming of HIV-infected individuals reverses the immune response to normal, leads to a decline in HIV-1 viral loads and the expression on the surface of CD4^+^ T lymphocytes of co-receptors responsible for cell to cell transmission of HIV-1, and an increase in CD4^+^ T lymphocyte numbers [103-106,123-127]. Thus, identification of individuals co-infected with HIV-1 and *S. mansoni* at very early stages for early de-worming, even if it only results in small reductions in viral load, may have benefits in delaying the progression or decrease the spread of the disease and importantly, may delay an individual’s need to begin anti-retroviral treatment (ARV) [126].

Despite the evidence that praziquantel has effects on some of the HIV-1 outcome parameters, there are still a number of issues that remain unsolved and will require further studies to investigate the exact mechanism of interaction between helminth and HIV-1 infections and the impact of anthelminthic treatment. The majority of the previous studies on this topic, were almost all cross-sectional in design, and some had significant limitations, including the interpretation of the results, the lack of comparison groups, short follow-up periods and small population sample sizes [91]. These limitations could in part be the cause of discrepancies between results: while some studies agree with the hypothesis that anthelminthic treatment either with albendazole (for gut nematodes), diethylcarbamazine (for filarial worms) or praziquantel, have positive effects on HIV-1 parameters, other studies do not agree with this observation. As yet, there is insufficient data to show clearly that de-worming of HIV-1 co-infected individuals has a beneficial effect on HIV-1 viral loads and CD4^+^ lymphocytes. As suggested by other authors, longitudinal studies exploring the interactions of HIV-1 and *S. mansoni* infections are warranted. These studies should focus on understanding the effects of the intensity of *S. mansoni* infection on HVI-1 viral loads and CD4^+^ T lymphocytes and on the development of hepatosplenic pathologies in co-infected individuals. The assessment of the impact of anthelminthic treatment on HIV-1 parameters should be made a priority.

## Conclusion

In Sub-Saharan Africa, HIV-1 and *S. mansoni* infections are co-endemic and co-infection occurs in highly endemic areas. There is evidence to support the occurrence of interactions between the two infections in a single co-infected human host. Furthermore, published data supports the hypothesis that helminth infections can influence important parameters of HIV infection such as CD4^+^ lymphocyte counts and HIV viral loads. Thus, in areas of co-endemicity, it is important to integrate control programmes for HIV-1 and schistosomiasis to give an opportunity for early identification of co-infected individuals and provide an opportunity for early deworming to reduce the fast progression and transmission of HIV-1.

Lastly, the available evidence on the interactions of HIV-1 and *S. mansoni* and the impact of praziquantel treatment on CD4^+^ lymphocytes and HIV-1 viral loads remains inconclusive and does call for further field studies to resolve discrepancies. Large randomized controlled trials with longer follow-up periods are required in order to assess the interactions between *S. mansoni* and HIV-1 and the impact of deworming cycles on the HIV-1 progression in populations constantly exposed to *S. mansoni* infection and at high risk of HIV infection.

## Competing interest

HDM is supported by the Training Health Researchers into Vocational Excellence in East Africa (THRiVE) Programme funded by Wellcome Trust, grant number 087540. The authors declare that there have no competing interest.

## Authors’ contributions

HDM designed the study, searched for literature and prepared the first draft of the manuscript, FN, SW, SMK, DM, RW, JH and DWD critically reviewed the manuscript. All authors have read and approved the final manuscript.

## Supplementary Material

Additional file 1 Multilingual abstracts in the six official working languages of the United Nations. Click here for file
