# Supplementary material for: Epidemiology and interactions of Human Immunodeficiency Virus – 1 and Schistosoma mansoni in sub-Saharan Africa
Source: Infect Dis Poverty. 2013 Jan 24;2:2. doi: 10.1186/2049-9957-2-2 (PMC3707091; doi:10.1186/2049-9957-2-2)
Supplement: Additional file 1 — Multilingual abstracts in the six official working languages of the United Nations. [file 2049-9957-2-2-S1.pdf]

Tanslation of the abstract into the six official working languages of the United Nations

علم الأوبئة وتفاعلات فيروس نقص المناعة -1 و بلهارسيا المجاري البولية في صحراء أفريقيا الجنوبية

هامفري د.مازيجو، فريد نواها، شونا ويلسون، سافاري م.كينونج هاي، دومينيكا مورونا، رباكا وايهنيا، جورج هويكل باش ، دافيد و. دون

مقدمة

ينشر فيروس نقص المناعة البشرية /الإيدز و بلهارسيا المجاري البولية في صحراء أفريقيا الجنوبية و تحدث العدوى المتزامنة كثيرا.

منذ أوائل التسعينيات كانت هناك اقتراحات ترجح أن العدوتان قد يتفاعلا مع بعضهما البعض ويحفزان آثار بعضهما داخل العائل البشري المصاب .

وبالطبع فقد كان اقتراح يرجح أن العدوى ببلهارسيا المجاري البولية قد تكون عاملا خطرا يحفز انتقال فيروس نقص المناعة وانتشاره في أفريقيا، و إذا كان ذلك صحيحا فسيعقب الطرد الكبير للديدان ظهور آثار مفيدة وجيدة على ديناميكية انتقال فيروس نقص المناعة.

يتغير علم أوبئة فيروس نقص المناعة البشرية في الدول الأفريقية لينتقل من المناطق الحضرية إلى المناطق الريفية حيث ينتشر بكثرة مرض بلهارسيا المجاري البولية وتنقص بشدة الخدمات الصحية العامة.

من ناحية أخرى ، تظل عملية ظهور مرض فيروس نقص المناعة البشرية / بلهارسيا المجاري البولية المتتالي غير معروفة، ونحن هنا نقدم أدلة عن علم أوبئة فيروس نقص المناعة البشرية / بلهارسيا المجاري البولية ، ونناقش العدوى المتزامنة وعلاقات الإصابات البيولوجية المحتملة بين العدوتين بهما و الآثار المحتملة للعلاج بعقار البرازكوانتيل على الحمولات الفيروسية لفيروس نقص المناعة البشرية ، أعداد خلايا $CD4^+$ و نسبة الخلايا اللمفاوية من نوعي $CD4^+/CD8^+$.

يوضح الإطار النظري للدراسة المتاح الذي نقدمة أن هناك أدلة تدعم افتراضية أن العدوى بمرض بلهارسيا المجاري البولية يمكن أن يؤثر على تضاعف أعداد فيروس نقص المناعة البشرية ، و انتقاله من خلية لأخرى، وانتشار الإصابة بفيروس نقص المناعة البشرية كما يقاس بأعداد خلايا $CD4^+$ T المتناقصة . إذا كان الأمر كذلك فطرد الديدان من الأشخاص الحاملين لفيروس نقص المناعة البشرية الذين يعيشون في المناطق توطن المرض قد تؤثر على الأحمال الفيروسية لفيروس نقص المناعة البشرية و أعداد خلايا $CD4^+$ T.

Translated from English version into Arabic by Einass, through

TRANSLATORS
WITHOUT BORDERS

在撒哈拉以南非洲地区人类免疫缺陷病毒-1 和曼氏血吸虫的流行病学和相互作用

Humphrey D. Mazigo, Fred Nuwaha, Shona Wilson, Safari M. Kinung'hi, Domenica Morona, Rebecca Waihenya, Jorg Heukelbach, David W. Dunne

摘要

HIV-1/AIDS 和曼氏血吸虫广泛分布于撒哈拉以南非洲地区，且常见合并感染。自 20 世纪 90 年代初，认为在合并感染 HIV-1 和曼氏血吸虫的人体中，它们相互作用，且可能互相影响。事实上，在非洲，曼氏血吸虫感染已被认为是 HIV 传播和发展的一个危险因素。如果确实如此，进行群体驱虫可能会对 HIV 的传播动力学产生有利影响。非洲国家 HIV 的流行病学特征正在发生变化，从城市转移到曼氏血吸虫病高发和缺乏公共卫生服务的农村。然而，HIV-1/曼氏血吸虫合并感染的发病机制尚不清楚。本文阐述了 HIV-1/曼氏血吸虫的流行病学，讨论了合并感染和两种感染之间可能的生物因果关系，以及吡喹酮治疗对 HIV-1 病毒载量、CD4$^+$计数和 CD4$^+$/CD8$^+$比值的影响。现有文献表明，有证据支持以下假设，即曼氏血吸虫感染会影响 HIV-1 病毒的复制，细胞间的传输，以及因 CD4$^+$ T 淋巴细胞数降低而增加 HIV 病毒数。如果这样的话，在流行地区对 HIV 阳性者进行驱虫可能会影响 HIV-1 病毒载量和 CD4$^+$ T 淋巴细胞数。

Translated from English version into Chinese by Yang Pin, through

CHINESE CENTER FOR DISEASE CONTROL AND PREVENTION
NATIONAL INSTITUTE FOR PARASITIC DISEASES

Épidémiologie et interactions du virus de l'immunodéficience humaine-1 et *Schistosoma mansoni* en Afrique Subsaharienne

Humphrey D. Mazigo, Fred Nuwaha, Shona Wilson, Safari M. Kinung'hi, Domenica Morona, Rebecca Waihenya, Jorg Heukelbach, David W. Dunne

RÉSUMÉ DE SYNTHÈSE :

La co-infection survient fréquemment entre les virus de l'immunodéficience humaine-1/SIDA et *Schistosoma mansoni* qui sont répandus en Afrique subsaharienne. Depuis le début des années 1990, il a été suggéré que les deux infections peuvent interagir et potentialiser les effets l'une de l'autre chez les hôtes humains co-infectés. En effet, l'infection par le *S. mansoni* a été proposée comme facteur de risque de transmission et de progression du VIH en Afrique. Si tel est le cas, il s'ensuivrait qu'une vermifugation massive pourrait avoir des effets bénéfiques sur la dynamique de transmission du VIH-1. L'épidémiologie du VIH dans les pays africains est en train de changer, passant des zones urbaines vers les zones rurales où le taux de prévalence du *Schistosoma mansoni* est élevé et où les services de santé publique sont insuffisants. D'un autre côté, la pathogenèse de la co-infection VIH-1/*S. mansoni* qui en découle reste inconnue. Nous proposons ici un compte rendu de l'épidémiologie du VIH-1 et du *S. mansoni*, discutons de la co-infection et des relations biologiques de causalité possibles entre les deux infections. Nous abordons également l'impact potentiel d'un traitement par praziquantel des charges virales du VIH-1, des taux de $CD4^{+}$ et du rapport $CD4^{+}/CD8^{+}$. Notre examen de la littérature disponible indique qu'il existe des éléments de preuve à l'appui de l'hypothèse selon laquelle les infections par *S. mansoni* peuvent influer sur la réplication du VIH-1 et la transmission de cellule à cellule, ainsi que sur l'accroissement de la progression du VIH, mesurée par la chute des numérations des lymphocytes T-CD4+. Dans l'affirmative, la vermifugation des sujets VIH positifs vivant dans les zones d'endémie pourrait avoir un impact sur la charge virale du VIH-1 ainsi que sur les numérations des lymphocytes T-CD4+.

Translated from English version into French by Lola Cello-Kirkham, through

TRANSLATORS
WITHOUT BORDERS

Эпидемиология и взаимодействие вируса иммунодефицита человека -1 и *Schistosoma mansoni* (шистосомы Мансона) в странах Африки, расположенных к югу от Сахары

Хамфри Ди Маджио, Фред Нуваха, Шона Уилсон, Сафари М. Кинунг'хи, Доменика Морона, Ребекка Уэхения, Йорг Хакельбах, Девид В. Данне

Аннотация

Вирус иммунодефицита человека-1/СПИД и *Schistosoma mansoni* широко распространены в странах Африки, расположенных к югу от Сахары, и очень часто имеет место совместное инфицирование. С начала 90-х годов предполагалось, что две эти инфекции могут взаимодействовать и потенцировать действие друг друга в организмах людей-носителей, зараженных обеими. Более того, инфекция *S. Mansoni* считалась фактором риска для передачи и прогрессирования ВИЧ в Африке. В таком случае, массовая дегельментизация позитивно повлияла бы на динамику передачи ВИЧ-1. Эпидемиология ВИЧ в странах Африки меняется, переходя от городских к сельским районам, где широко распространена *Schistosoma mansoni* и существует дефицит услуг общественного здравоохранения. С другой стороны, последовательный патогенезис совместного инфицирования ВИЧ-1/*S. mansoni* остается неизвестным. Тут мы изложили эпидемиологию ВИЧ-1 и *S. mansoni*, обсуждаем совместное инфицирование и возможную биологическую причинно-следственную связь между этими инфекциями, а также потенциальное влияние лечения празиквантелом на вирусные нагрузки ВИЧ-1, количество $CD4^+$ и соотношение $CD4^+/CD8^+$. Наш обзор доступной литературы указывает на существование признаков, подтверждающих гипотезу, состоящую в том, что инфекции *S. mansoni* могут влиять на репликацию ВИЧ-1, передачу от клетки клетке, а также увеличение прогрессии ВИЧ, измеряющуюся уменьшением количества $CD4^+$ Т-лимфоцитов. Если это так, дегельминтизация ВИЧ-позитивных людей, живущих в эндемических областях, может повлиять на вирусную нагрузку HIV-1 и количество $CD4^+$ Т-лимфоцитов.

Translated from English version into Russian by Kateryna Moroz, through

TRANSLATORS
WITHOUT BORDERS

Epidemiología e interacciones del virus de Inmunodeficiencia Humana-1 y *Schistosoma mansoni* en África subsahariana

Humphrey D. Mazigo, Fred Nuwaha, Shona Wilson, Safari M. Kinung'hi, Domenica Morona, Rebecca Waihenya, Jorg Heukelbach, David W. Dunne

RESUMEN

El Virus de Inmunodeficiencia Humano-1/SIDA y el *Schistosoma mansoni* son endémicos en África subsahariana y la infección concomitante es muy común. Desde principios de la década de 1990, se ha sugerido que las dos infecciones pueden interactuar y potenciar sus efectos mutuamente en huéspedes humanos con ambas infecciones. Efectivamente se ha sugerido que la infección por *S. mansoni* constituye un factor de riesgo para la transmisión y progresión de VIH en África. Si es así, se esperaría que una desparasitación masiva pudiera ser beneficiosa en relación a la dinámica de la transmisión del VIH-1. La epidemiología del VIH en países africanos está cambiando, trasladándose de zonas urbanas a zonas rurales donde la prevalencia del *Schistosoma mansoni* es alta y los servicios de salud pública son deficientes. Por otro lado, la consecuencia de la patogénesis de la infección concomitante del VIH-1/*S. mansoni* se desconoce. Aquí entregamos una descripción de la epidemiología del VIH-1 y el *S. mansoni*, discutimos la coinfección y posible relación causal biológica entre las dos infecciones y el impacto potencial del tratamiento con praziquantel sobre la carga viral de VIH-1, recuento de $CD4^+$ y relación $CD4^+/CD8^+$. Nuestra revisión de la literatura disponible indica que no hay evidencia que apoye la hipótesis de que las infecciones por *S. mansoni* pueden influenciar la replicación del VIH-1, transmisión de célula a célula, como tampoco una progresión aumentada del VIH medida por recuentos disminuidos de linfocitos T $CD4^+$. Si es así, entonces la desparasitación de individuos VIH positivo que viven en áreas endémicas podría tener un impacto sobre la carga viral de VIH-1 y los recuentos de linfocitos T $CD4^+$.

Translated from English version into Spanish by Claudia, through

TRANSLATORS
WITHOUT BORDERS
